# Phenotypic and Functional Analyses of B7S1 in Ovarian Cancer

**DOI:** 10.3389/fmolb.2021.686803

**Published:** 2021-07-09

**Authors:** Dongli Cai, Fang Wang, Changgang Wang, Liping Jin

**Affiliations:** ^1^ Clinical and Translational Research Center, Shanghai First Maternity and Infant Hospital, School of Medicine,Tongji University, Shanghai, China; ^2^ Department of Gynaecology, Shanghai East Hospital, School of Medicine,Tongji University, Shanghai, China; ^3^ Department of General Surgery, Ruijin Hospital, Shanghai Jiao Tong University School of Medicine, Shanghai, China

**Keywords:** ovarian cancer, immune checkpoint, B7S1, tumor-infiltrating lymphocytes, immunotherapy

## Abstract

**Background:** Although programmed death (PD) ligand 1 (PD-L1)/PD-1 inhibitors show potent and durable antitumor effects in a variety of tumors, their efficacy in patients with OvCa is modest. Thus, additional immunosuppressive mechanisms beyond PD-L1/PD-1 need to be identified.

**Methods:** The mRNA expression profiles of OvCa patients were obtained from The Cancer Genome Atlas (TCGA) database. The expression and clinical characteristics of *VTCN1* (encoding B7S1) in OvCa were analyzed. The molecular interaction network, Gene Ontology (GO) analysis and Gene set enrichment analysis (GSEA) were used to functionally annotate and predict signaling pathways of *VTCN1* in OvCa. Moreover, 32 treatment-naïve patients with OvCa were recruited to assess B7S1 expression. The cytotoxic immune phenotypes in distinct subgroups were analyzed.

**Results:** B7S1 expression was increased in tumor sections compared with that in normal tissues from OvCa patients at both the mRNA and protein levels. *VTCN1* expression was significantly correlated with the mRNA expression levels of several other co-inhibitory immune checkpoints. B7S1 protein was found to be highly expressed in CD45^+^CD68^+^ myeloid cells, whereas its putative receptor was expressed in CD8^+^ tumor-infiltrating lymphocytes (TILs). Furthermore, expression of B7S1 in antigen-presenting cells (APCs) was significantly correlated with the cytolytic function of CD8^+^ TILs. Functional annotations indicated that *VTCN1* was involved in regulating T cell-mediated immune responses and participated in the activation of a variety of classic signaling pathways related to the progression of human cancer.

**Conclusion:** In OvCa, B7S1 was highly expressed and may initiate dysfunction of CD8^+^ TILs, which could be targeted for cancer immunotherapy.

## Introduction

Ovarian cancer (OvCa) is one of the most lethal gynecologic malignancies, with increasing global incidence. Global Cancer Statistics estimated that over 295,414 new OvCa cases and 184,799 OvCa-related deaths occurred worldwide in 2018 (Bray et al., 2018). Owing primarily to the lack of effective screening strategies and the absence of early specific symptoms, more than 75% patients with OvCa present disease stage III or IV at diagnosis (Hung et al., 2016), thereby contributing to a poor overall survival. Although aggressive frontline treatments with taxane/platinum-based chemotherapy and cytoreductive surgery, more than 70% of patients with advanced-stage cancer relapse within 5 years and become resistant to chemotherapy; the 5-year overall survival rate remains ominously low at 45% (Capriglione et al., 2017). Thus, there is an urgent need for the development of novel therapeutics for advanced OvCa.

Recent studies have shown that, like many solid tumors, OvCa is immunogenic (Zhang et al., 2003) and can elicit a spontaneous antitumor immune response (Cai et al., 2020). Engaging the immune system is a critical component of optimal OvCa therapy, and the quality of tumor-infiltrating T cells (TILs) is a critical determinant of outcomes in patients with OvCa (Zhang et al., 2003; Hamanishi et al., 2007; Hwang et al., 2012). T cell-mediated immune responses are regulated by several costimulatory and co-inhibitory signals (Sun et al., 2018). Evidence in several cancer systems has shown that inhibitory immune checkpoint receptors expressed on T cells promote T-cell exhaustion, dampen host immunity, and assist tumor evasion (Thommen et al., 2018). Blockade of these inhibitory checkpoint receptors with specific antibodies has been reported to reinstate antitumor responses at different levels (Pardoll, 2012).

Programmed death (PD)-1 is a prototypical co-inhibitory checkpoint receptor that has emerged as a critical intrinsic modulatory mechanism for impairing natural antitumor immunity (Gaillard et al., 2016). Interfering with PD-1 or its ligand PD-L1 has been shown to enhance antineoplastic immune responses through the recovery of T-cell function in a wide spectrum of tumors (Mariathasan et al., 2018), including melanoma (Patel and Kurzrock, 2015), non-small cell lung cancer (Patel and Kurzrock, 2015) and renal cell carcinomas (McDermott et al., 2016). However, although the PD-1/PD-L1 pathway blockade produces durable clinical responses in preclinical tests for OvCa, the best overall response rate in 26 PD-L1-positive patients treated with pembrolizumab (anti-PD-1) was only 11.5% (Disis et al., 10718). Since immune checkpoint molecules function nonredundantly and cooperatively to fine-tune immune responses and promote T-cell exhaustion (Li et al., 2018), additional immunosuppressive mechanisms need to be identified.

B7S1 (VTCN1, B7-H4, B7x) is a member of the B7 superfamily and shows 25% amino acid identity with other B7 family members (Hansen et al., 2009). B7S1 mRNA is broadly expressed; however, the restricted pattern of protein expression in normal tissue suggests post-transcriptional regulation (Choi et al., 2003). Aberrant B7S1 expression has been reported in a vast majority of human malignancies, including melanoma (Quandt et al., 2011), renal cell carcinoma (Krambeck et al., 2006), breast cancer (Tringler et al., 2005), non-small cell lung cancer (Sun et al., 2006), thyroid cancer (Zhu et al., 2013), and hepatocellular carcinoma (Kang et al., 2017). B7S1 expression in multiple solid tumors is positively correlated with malignant phenotypes, such as advanced lymph node metastasis, high tumor stage, poor differentiation, early recurrence, and is reversely related to the infiltration intensity of TILs, as well as the overall survival rate (Wang and Wang, 2020).

B7S1 expression is restricted to antigen-presenting cells (APCs), and its putative receptor is induced on activated T cells to inhibit their proliferation, cytokine production, and cytotoxicity (Kryczek et al., 2006; Kryczek et al., 2007; Li et al., 2018). In human hepatocellular carcinoma, B7S1 expression is reportedly upregulated on APCs, and its putative receptor is co-expressed with PD-1 on activated early CD8^+^ TILs, to promote T cell exhaustion and depress anti-tumor immune response via upregulating Eomes (Li et al., 2018). Studies have shown that B7S1 is closely relevant to classical signaling pathways. In esophageal squamous cell carcinoma, by activating JAK2/STAT3 pathway, B7S1 stimulated the secretion of IL-6, which in turn upregulated the expression of B7S1, thereby mutually enhancing the growth and tumorigenicity of cancer cells (Chen et al., 2016). Incubated T cells with B7S1-Ig fusion protein *in vitro* significantly inhibits the proliferation and cytotoxic activity of T cells, by interfering with the activation of ERK, JNK, and AKT (Wang et al., 2012; Wang and Wang, 2020). B7S1 silencing enhanced drug-induced apoptosis by inhibiting the PTEN/PI3K/AKT pathway in triple-negative breast cancer cells (Wang et al., 2018). Furthermore, blockade of B7S1 down-regulated the transcription of CXCL12/CXCR4. By activating ERK1/2, AKT, PI3K and the other signaling pathways, CXCL12/CXCR4 axis is widely involved in the proliferation, invasion and metastasis of tumor cells (Peng et al., 2015).

B7S1 is highly expressed in primary and metastatic serous, endometrioid, clear cell and epithelial ovarian carcinomas, but is low in mucinous and non-epithelial ovarian carcinomas (Wang and Wang, 2020). According to a recent study, B7S1 was predominantly expressed by ovarian cancer cells, and this alteration is positively correlated with the expression of C-X-C motif chemokine ligand 17 and the proportion of infiltrating mature APCs (MacGregor et al., 2019). However, in 2006, a suppressive macrophage population with B7S1 expression has been detected in human ovarian carcinoma. The B7S1^+^ macrophages express CD86 at a higher level and have stronger inhibitory activity than B7S1^−^ macrophages (Kryczek et al., 2006; Kryczek et al., 2007). Due to high expression level of B7S1 in OvCa was found significantly associated with tumor stage (Liang et al., 2016) and a worse progression-free survival (PFS) (Ye et al., 2018), B7S1 may serve as a promising candidate target for OvCa immunotherapy.

Accordingly, in this study, we aimed to determine the expression and roles of B7S1 in OvCa, with a focus on its relation to cancer-associated immune responses. Our findings demonstrated that B7S1 suppressed antitumor immunity and supported the applications of B7S1 as a promising target for immunotherapy in OvCa.

## Materials and Methods

### Analysis of the Public Dataset

RNA sequencing-based gene expression data of samples from patients with OvCa were obtained from Gene Expression Profiling for Interactive Analysis (GEPIA) for Cancer Genomics (http://gepia.cancer-pku.cn/) (Tang et al., 2017) and TISIDB (http://cis.hku.hk/TISIDB/) (Ru et al., 2019).

### Human Specimens

Fresh tumor tissues, malignant ascites, and matched blood were collected from 32 patients with OvCa undergoing primary surgical treatment without chemotherapy at Shanghai First Maternity and Infant Hospital. All experimental protocols were approved by the Ethical Committee of the Shanghai First Maternity and Infant Hospital (IEC approval NO. 2017-100), and informed consent was obtained from patients prior to their enrollment in the study.

### Isolation of Peripheral Blood Mononuclear Cells and TILs from Tumors or Ascites

Blood and ascites from patients with OvCa were drawn into heparinized tubes and centrifuged on Ficoll-Hypaque density gradients (cat. no. 17-1440-02; GE Healthcare Life Sciences). Fresh tumor tissues from patients with OvCa were digested in RPMI-1640 medium supplemented with 1 mg/ml collagenase IV (cat. no. 17104019; Gibco) for 30 min at 37°C prior to Ficoll-Hypaque density gradient centrifugation. This method has been described previously (5).

### Immunofluorescence

Paraffin sections of human OvCa specimens were dewaxed in xylene, dehydrated in ethanol, subjected to heat-induced epitope retrieval, and then incubated with primary antibodies against human CD45 (cat. no. ab10559; Abcam, Cambridge, United Kingdom) and B7S1 (MIH43; cat. no. ab110221; Abcam, Cambridge, United Kingdom) at 4°C overnight. AffiniPure F (ab’)2 Fragment donkey anti-rabbit immunoglobulin (cat. no. 711-546-152; Jackson Immuno Research, West Grove, PA, United States) and AffiniPure F (ab’)2 Fragment donkey anti-mouse immunoglobulin (cat. no. 715-166-150; Jackson Immuno Research, West Grove, PA, United States) were chosen as the secondary antibodies. All slides were incubated with mounting medium containing 4′,6-diamidino-2-phenylindole for 20 min. Images were obtained using a Zeiss fluorescence microscope. Quantification analysis was performed using ImageJ software (National Institutes of Health, Bethesda, MD, United States). The method has been described previously (Cai et al., 2020).

### Flow Cytometry

The following fluorescent dye-conjugated antibodies were used: anti-CD45-PerCP-CY5.5 (HI30; cat. no.304028, Biolegend), anti-lineage-fluorescein isothiocyanate-FITC (UCHT1, HCD14, 3G8, HIB19, 2H7, HCD56; cat. no. 348701, Biolegend), anti-human leukocyte antigen (HLA)-DR-AF700 (L243; cat. no. 307626Biolegend), anti-CD14-allophycocyanin (APC)-CY7 (M4P9; cat. no. 557831, BD), anti-CD15-phycoerythrin (PE)-CY5 (W6D3; cat. no. 323014, Biolegend), anti-B7S1-PE-CY7 (MIH43; cat. no. 358106, Biolegend), anti-CD3-PerCP-CY5.5 (OKT3; cat. no. 300328, Biolegend), anti-CD56-FITC (HCD56; cat. no. 318304, Biolegend), anti-CD4-PE-CF594 (L200; cat. no. 562402, BD), anti-CD8-AF700 (SK1; BD), anti-FoxP3-PE-CF594 (259D/C7; cat. no. 563955, BD), anti-T cell immunoglobulin and mucin domain-containing protein 3 (TIM-3)-PE (F38-2E2; cat. no. 345006, Biolegend), anti-CD27-PerCP-CY5.5 (O323; cat. no. 302820, Biolegend), anti-Ki-67-AF700 (B56; BD), anti-tumor necrosis factor (TNF)-α-PE-CF594 (Mab11; cat. no. 502946, Biolegend), and anti-interferon (IFN)-γ-PE-CY7 (B27; cat. no. 506518, Biolegend). Dead cells were excluded by viability dye staining (Fixable viability dye eF506; cat. no. 65-0866-18, eBioscience), as described previously (Cai et al., 2020).

To detect the expression of human B7S1 receptor, cell suspensions were incubated with 10 μg/ml biotin-labeled human B7S1-mouse IgG2a Fc fusion protein (generated in-house) at 4°C for 40 min and then with 0.5 μg/ml streptavidin Brilliant Violet 421 (cat. no. 405225 Biolegend) together with surface antibodies. Cells were acquired using an LSRFortessa flow cytometer, and data were analyzed using FlowJo.X software (Tree Star, Ashland, OR, United States).

### Protein–Protein Interaction (PPI) Network Construction

The STRING (https://string-db.org/) online database was used to analyze the functional interactions between proteins. Cytoscape (version 3.5) was employed to visualize the molecular interaction network.

### Functional Annotations

mRNA expression profiles of OvCa were obtained from The Cancer Genome Atlas (TCGA, https://cancergenome.nih.gov/) online database. Gene Ontology (GO) enrichment analysis for hub genes was performed and visualized using ClueGo (version 2.5.7) and CluePedia (version 1.5.7). Gene set enrichment analysis (GSEA) was used to predict potential hallmarks of *VTCN1* in OvCa.

### Statistics

Statistical analyses were conducted with Prism 6.0 software (GraphPad), using the appropriate tests as indicated in the legends. All values are expressed as means ± standard errors of the means. Results with *p* values less than 0.05 were considered statistically significant.

## Results

### *VTCN1* was Highly Expressed in Human OvCa and was Correlated With Multiple Co-inhibitory Immune Checkpoint Genes

**FIGURE 1:**
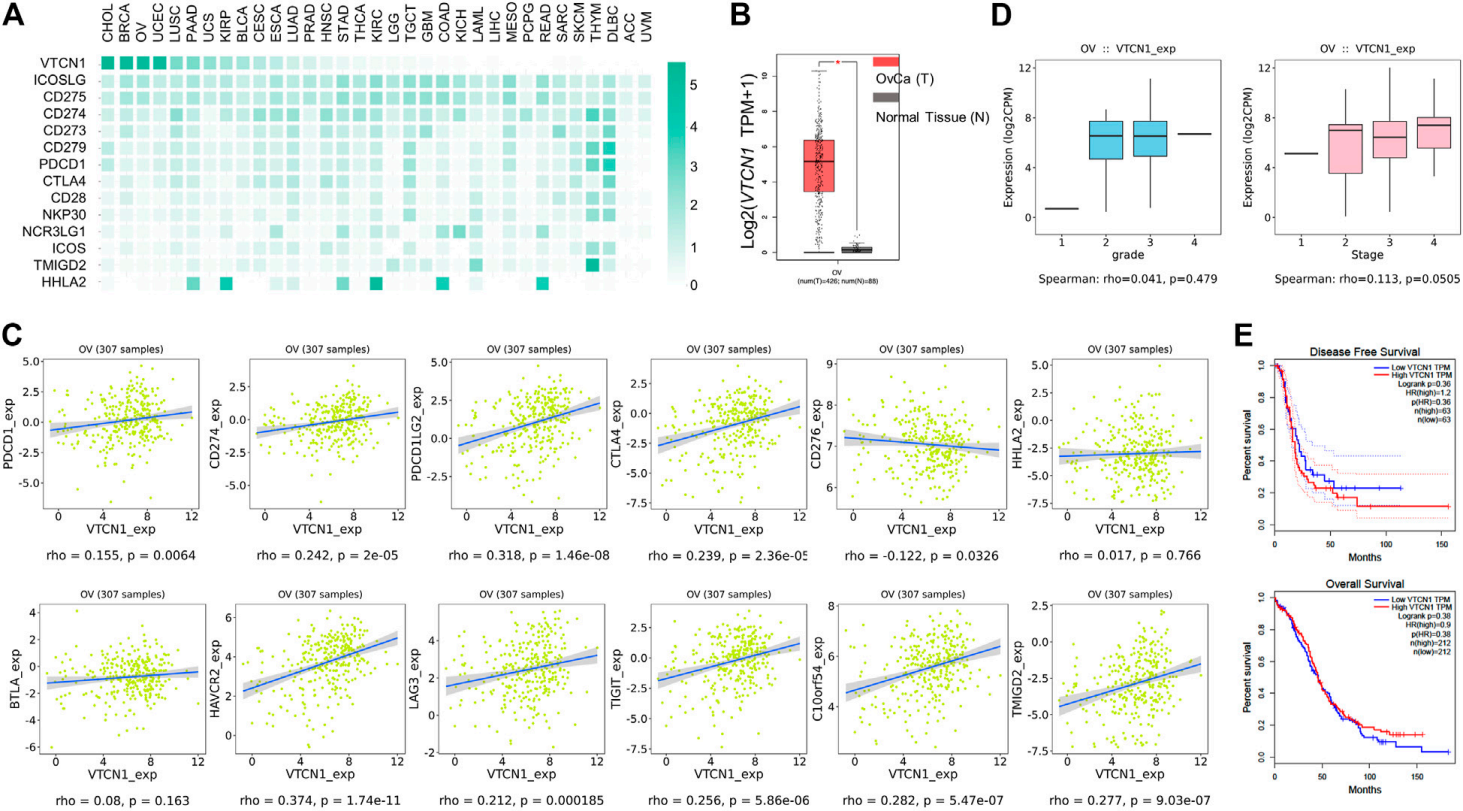
*VTCN1* was overexpressed in OvCa and correlated with multiple co-inhibitory immune checkpoint genes. **(A)** Heatmap analysis of the mRNA expression of B7/CD28 family genes in various human tumors, shown as scaled log2-fold changes (GEPIA data). **(B)** The expression levels of *VTCN1* in human OvCa and normal controls. The data were derived from TCGA database and are shown on a log2 (TPM +1) scale. TPM: transcripts per million. The *p* value cutoff was 0.01. *p < 0.05. **(C)** Correlations of *VTCN1* expression with tumor grade (left) and stage (right) in patients with OvCa. **(D)** Correlations of *VTCN1* expression with the mRNA expression levels of several immune checkpoint proteins. **(E)** Correlations of *VTCN1* expression with disease-free survival (upper panel) and overall survival (bottom panel) in patients with OvCa.

The low response rate to PD-1 blockade in OvCa may be associated with co-expression of other co-inhibitory immune checkpoint molecules in the tumor microenvironment (TME). To identify potential targets in patients with OvCa, we examined the mRNA expression levels of several checkpoint molecules in various tumors. RNA-sequencing data from TCGA revealed high upregulation of *VTCN1* (encoding B7S1) in several solid tumors, including human OvCa, as compared with known B7/CD28 family members (Figure 1A). Upregulation of *VTCN1*, i.e., a log_2_ (TPM+1) fold increase of more than 32, was observed in OvCa compared with normal tissues (Figure 1B). Moreover, the expression of *VTCN1* was significantly positively correlated with the expression of some other checkpoint genes, such as *CTLA4*, *HAVCR2*, *LAG3*, *TIGIT*, and *C10orf54* (Figure 1C). In addition, *VTCN1* expression was not significantly correlated with tumor grade or tumor stage (Figure 1D). Stratification of patients with OvCa by *VTCN1* expression revealed no significant differences in disease-free survival or overall survival, although patients with high *VTCN1* expression had a high hazard ratio (HR; HR = 1.2) for relapse (Figure 1E). The highly expressed B7S1 in OvCa has been reported significantly associated with tumor stage (Liang et al., 2016) and a worse progression-free survival (Ye et al., 2018). The inconsistency between mRNA level and protein level may be related to the complex mechanism of post-transcriptional regulation. Taken together, these results implied that B7S1 may contribute to antitumor immunosuppression in OvCa.

### B7S1 Protein Was Mainly Expressed by Intratumoral Myeloid Cells in Human OvCa

**FIGURE 2:**
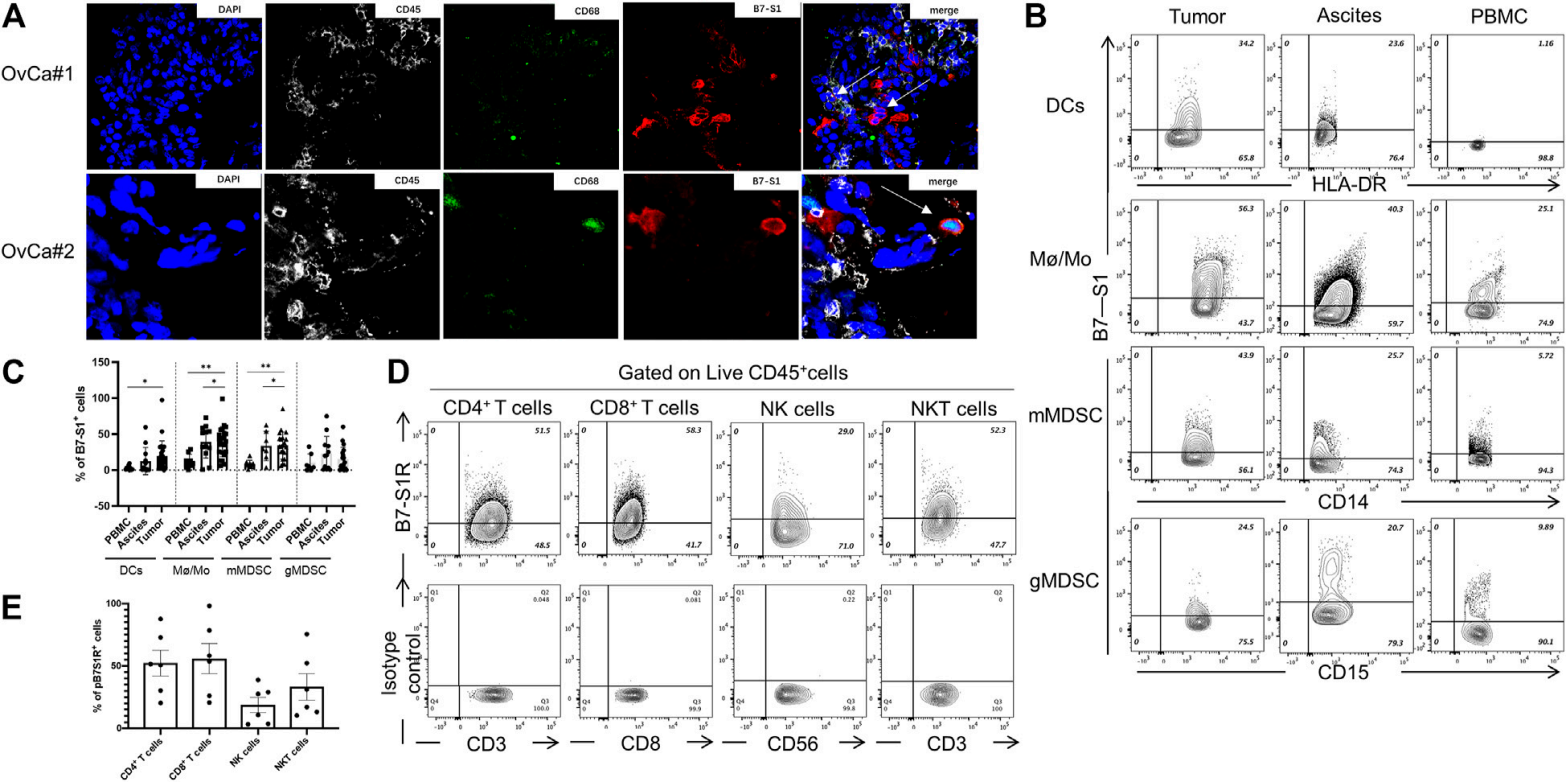
B7S1 protein was mainly expressed in intratumoral myeloid cells in human OvCa. **(A)** Representative immunofluorescence images of B7S1 in human OvCa. Original magnification: 40x (OvCa#1), 100x(OvCa#2). **(B,C)** Representative figures and summarized data showing the percentages of B7S1-positive DCs, Mø/Mo, mMDSCs, and gMDSCs in tumors, ascites, and PBMCs from patients with OvCa. Statistical analysis was performed using one-way analysis of variance followed by Tukey’s multiple comparison tests. The data are presented as means ± standard errors of the means; ^ns^ *P* > 0.05, *p < 0.05, **p < 0.01, and ***p < 0.001. **(D,E)** Representative figures and summarized data of pB7S1R expression on CD4^+^ T cells, CD8^+^ T cells, NK cells, and NK T cells in OvCa tumors detected by a biotinylated hB7S1-mIgG2a Fc fusion protein.

Because *VTCN1* is differentially expressed in OvCa subtypes (Chen et al., 2018), we next evaluated the protein levels of B7S1 in tumor samples from primary debulking surgeries of 32 treatment-naïve patients with epithelial OvCa (Table. 1) by immunofluorescence and flow cytometry (Cai et al., 2020). Compared with normal ovary tissues, B7S1 was upregulated in tumor sections, as detailed in our previous study (Cai et al., 2020). Contrary to reported data demonstrating that B7S1 expression is restricted to the tumor cell compartment (MacGregor et al., 2019), our study showed that B7S1 was expressed on both CD45^−^ and CD45^+^ cells (Figure 2A, indicated by the white arrows). In CD45^+^ cells, B7S1 was predominantly detected within the CD68^+^ myeloid compartment (Figure 2A).

**TABLE 1:** Clinical and pathological characteristics of patients with OvCa.

Patient no	Age	FIGO Stage	Pathology	Chemotherapy
P#1	32	IV	Low-grade adenocarcinoma	No
P#2	68	IIIc	Low-grade adenocarcinoma	No
P#3	59	IIIc	Endometrioid adenocarcinoma	No
P#4	55	IIIb	Low-grade adenocarcinoma	No
P#5	50	IIIc	Low-grade adenocarcinoma	No
P#6	42	IV	Serous papillary adenocarcinoma	No
P#7	54	IIc	Endometrioid adenocarcinoma	No
P#8	63	IIa	High-grade adenocarcinoma	No
P#9	28	IIIc	Mucinous adenocarcinoma	No
P#10	51	Ic	Adenocarcinoma	No
P#11	54	IIa	Endometrioid adenocarcinoma	No
P#12	45	Ic	Adenocarcinoma	No
P#13	67	IIIa	High-grade adenocarcinoma	No
P#14	48	IIIc	High-grade adenocarcinoma	No
P#15	49	IIIc	High-grade adenocarcinoma	No
P#16	54	IIIc	Low-grade adenocarcinoma	No
P#17	61	IIa	Endometrioid adenocarcinoma	No
P#18	60	-	Low-grade adenocarcinoma	No
P#19	67	IIb	High-grade adenocarcinoma	No
P#20	39	Ia	Mucinous carcinoma	No
P#21	66	IIb	High-grade adenocarcinoma	No
P#22	60	IIa	High-grade adenocarcinoma	No
P#23	48	Ib	Adenocarcinoma	No
P#24	61	IIIc	High-grade adenocarcinoma	No
P#25	55	-	Adenocarcinoma	No
P#26	51	IIc	Adenocarcinoma	No
P#27	-	-	High-grade serous carcinoma	No
P#28	67	IIb	Adenocarcinoma	No
P#29	49	IIIc	High-grade adenocarcinoma	No
P#30	51	IIIb	Low-grade adenocarcinoma	No
P#31	36	Ia	Clear cell carcinoma	No
P#32	46	IIc	High-grade serous carcinoma	No

Given that B7S1 could be detected on tumor-infiltrating CD45^+^ cells, we sought to identify which subsets of myeloid cells expressed this target. TILs from tumor tissues and tumor-associated lymphocytes from ascites were isolated by density-gradient centrifugation. PBMCs from the same patients were used as controls. B7S1 was predominantly expressed on myeloid dendritic cells (mDCs), CD14^+^HLA-DR^hi^ monocytes/macrophages (Mø/Mo), and CD14^+^HLA-DR^low/-^ monocytic myeloid-derived suppressed cells (mMDSCs). The percentages of B7S1-expressing APCs in the TME and ascites were significantly higher than those in peripheral blood (Figures 2B,C). We did not find a significant difference in B7S1 expression on CD15^+^ HLA-DR^low/-^ granulocytic myeloid-derived suppressed cells (gMDSCs) among tumors, ascites, and PBMCs (Figures 2B,C).

B7S1 is a negative immune checkpoint protein that binds to activated T cells and inhibits their proliferation and function (Li et al., 2018). To identify the potential targets of B7S1 in OvCa, we utilized a biotin-labeled human B7S1-mouse IgG2a Fc fusion protein to assess the expression of the putative B7S1 receptor (pB7S1R). pB7S1R was detected on CD4^+^ T, CD8^+^ T, natural killer (NK), and NK T cells in tumor tissues (Figures 2D,E). Because B7S1 expressed by tumor-infiltrating myeloid cells has been reported to induce dysfunction of antitumor CD8^+^ T cells in liver cancer (Li et al., 2018), the expression patterns of B7S1 and pB7S1R strongly suggested that B7S1 expression in OvCa may inhibit CD8^+^ T cell function.

### B7S1 Expression was Inversely Correlated With the Cytolytic Function of CD8^+^ TILs

**FIGURE 3:**
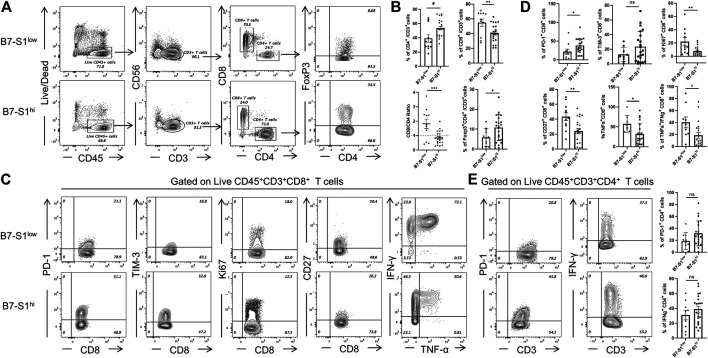
B7S1 expression was inversely correlated with the cytolytic function of CD8+ TILs. **(A,B)** Representative figures and summarized data of infiltrated T cells in tumor tissues of patients with OvCa in B7S1^hi^ and B7S1^low^ groups. **(C,D)** Representative figures and summarized data displaying PD-1, Ki67, CD27, TNFα, and IFNγ expression in CD8^+^ T cells in tumors. **(E)** Representative figures and summarized data displaying PD-1 and IFNγ expression in CD4^+^ T cells in tumors. The data are presented as means ± standard errors of the means; *p* value was computed by *t*-test. ^ns^ *P* > 0.05, *p < 0.05, **p < 0.01, and ***p < 0.001.

To investigate the relevance of B7S1 expression in the infiltration and function of CD8^+^ TILs, patients with OvCa were divided into two groups (B7S1^hi^ and B7S1^low^) based on the average frequency of B7S1^+^ cells in all CD14^+^ myeloid cells (Hong et al., 2019) (cutoff = 22.9%). Compared with B7S1^hi^ patients, significantly lower frequencies of CD4^+^ T cells and CD4^+^Foxp3^+^ cells were found in B7S1^low^ patients. In contrast, B7S1^low^ patients had markedly higher fractions of CD8^+^ TILs and CD8/CD4 ratios (Figures 3A,B). Moreover, CD8^+^ TILs isolated from B7S1^hi^ patients displayed the characteristic exhausted T-cell phenotypes, including higher levels of the co-inhibitory molecule PD-1, decreased surface expression of the co-stimulatory molecule CD27 and proliferation marker Ki67, and decreased levels of TNF-α upon phorbol myristate acetate/ionomycin stimulation in comparison with those from B7S1^low^ patients (Figures 3C,D). CD8^+^ TILs isolated from B7S1^hi^ patients tended to show increased TIM-3 expression (Figures 3C,D); the lack of a significant difference between the two groups may have been related to the small sample size in this study. In addition, although there was no statistically significant difference in the proportion of IFN-γ^+^ CD8 TILs (data not shown) between the two groups, which may be due to the lack of NK cells in some patients, the frequency of TNF-α^+^IFN-γ^+^ CD8 TILs was found to be increased in B7S1^low^ patients (Figures 3C,D). Since almost half of CD4^+^ T cells expressed pB7S1R as shown in Figure 1D, we also analyzed the expression of PD-1 and IFN-γ in CD4^+^ TILs. It was found that there were no obvious differences between B7S1^hi^ and B7S1^low^ groups (Figure 3E), which could be due to the limited sample size. Collectively, these results indicated that B7S1 expression on APCs was inversely correlated with the infiltration and cytolytic function of CD8^+^ TILs in OvCa.

### Functional Annotations and Predicted Signaling Pathways

**FIGURE 4:**
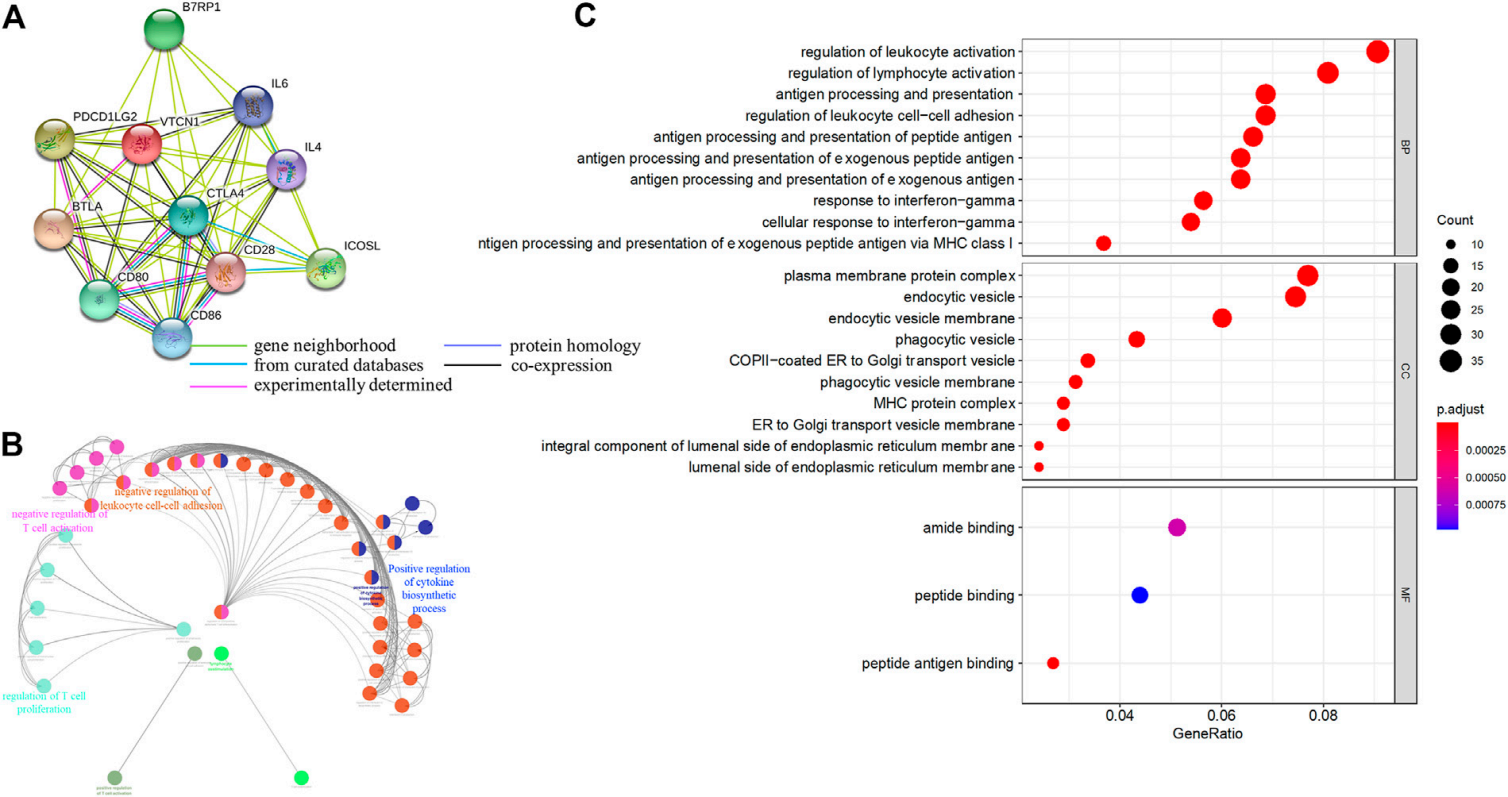
Functional annotations and predicted signaling pathway of *VTCN1* in OvCa. **(A)** The PPI network of *VTCN1* constructed using STRING. **(B)** Functional annotation of the *VTCN1*-related module genes constructed by ClueGo. **(C)** Significantly enriched GO annotations of *VTCN1* in OvCa. BP: biological processes. CC: cellular components; MF: molecular functions.

Next, we systematically analyzed the biological functions of *VTCN1* in OvCa. A PPI network of potential targets was constructed (Figure 4A), and the most significant modules were acquired using Cytoscape (Figure 4B). Functional analyses of *VTCN1* demonstrated that genes in this module were mainly enriched in the regulation of T-cell activation, T-cell proliferation, leukocyte cell-cell adhesion and cytokine biosynthetic process (Figure 4B). We selected genes that were co-expressed with *VTCN1* in OvCa from TCGA database, and the top 500 (*p* < 0.01 and correlation >0.3) positively and negatively correlated genes were processed *via* GO functional analysis using the cluster-filer package of R software (Yu et al., 2012).We found that the significant GO terms in the biological process category were tightly associated with the regulation of lymphocyte activation, antigen processing and presentation, and response to IFN-γ (Figure 4C).

**FIGURE 5:**
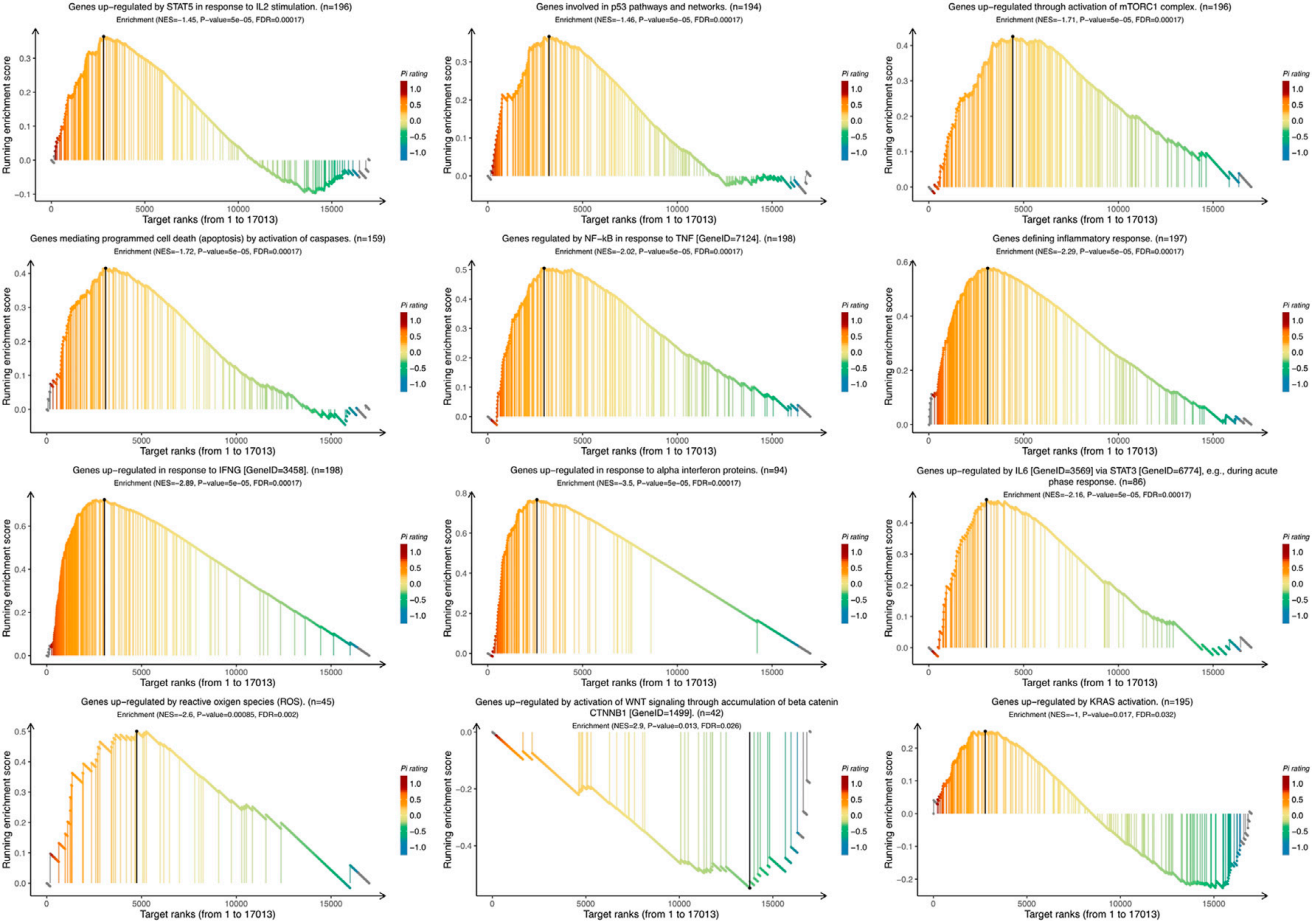
The Gene Set Enrichment Analysis (GSEA) of the relationship between the expression level of VTCN1 in the TCGA ovarian cancer dataset. The most involved significant hallmark pathways which were closely correlated with *VTCN1* in OvCa obtained by GSEA. NES: normalized enrichment score; FDR: false discovery rate.

To identify the *VTCN1*-related pathways activated in OvCa, we conducted GSEA between low and high *VTCN1* expression datasets. Significant differences (false-discovery rate <0.25, *p* < 0.05) in the enrichment of the Molecular Signature database Collection are shown in Figure 5. The results showed that *VTCN1* was mainly involved in IL-2/signal transducer and activator of transcription (STAT)5 signaling, p53 pathway, mammalian target of rapamycin complex 1 (mTORC1) signaling, apoptosis, TNF-α signaling via nuclear factor (NF)-κB, inflammatory response, IFN-γ response, IFN-α response, IL-6/Janus kinase (JAK)/STAT3 signaling, reactive oxygen species signaling, WNT/β-catenin signaling, and KRAS signaling. These results showed that high expression of *VTCN1* was closely associated with antitumor immune responses and malignancy in OvCa.

## Discussion

In this study, we demonstrated that B7S1 was highly expressed in OvCa tumor tissues compared with that in non-tumor tissues at both the mRNA and protein levels. B7S1 was mainly expressed by tumor-infiltrating APCs, and its putative receptor was expressed by CD8^+^ TILs in human OvCa. The expression of B7S1 in the TME was strongly correlated with poor CD8^+^ T-cell responses. Functional enrichment and GSEA analyses illustrated that *VTCN1* was significantly involved in T-cell regulation, cancer-related pathways, and hallmarks in OvCa. These results indicated that B7S1 could serve as a novel biomarker for diagnosis and could be considered as a potential immunotherapeutic target.

High expression of B7S1 in OvCa has been described, and the overexpression of B7S1 has been identified to be associated with tumor stage (Liang et al., 2016) and a worse progression-free survival (Ye et al., 2018). In our study, we further demonstrated robust expression of B7S1 in human OvCa. However, *VTCN1* expression was not significantly correlated with tumor grade and disease-free survival, an observation not consistent with protein levels, suggesting that it may be related to the mechanism of post-transcriptional regulation. In contrast to a previous study showing that B7S1 is predominantly expressed by tumor cells, we found that B7S1 could be detected in both CD45^−^ and CD45^+^ cells in the OvCa TME. In CD45^+^ cells, B7S1 was mainly detected within the CD68^+^ myeloid compartment, particularly mDCs, Mø/Mo, and mMDSCs. Moreover, the expression levels of B7S1 in tumor sections and ascites were significantly higher than those in PBMCs. According to previous reports (Motzer et al., 2015; Patel and Kurzrock, 2015; Yi et al., 2018; Cai et al., 2020), high PD-L1 expression in the TME is favorably correlated with increased response rates and clinical benefits in PD-L1/PD-1 blockade therapies. However, in OvCa, PD-L1 is observed rather low expressed on tumor cells, and the percentage of PD-L1-expressing APCs is not high in tumor sections or ascites (5). The low expression levels of PD-L1 may contribute to the low overall response rates in patients with OvCa after anti-PD-L1/PD-1 therapies. The high levels of B7S1 in the TME and the distinct expression patterns from PD-L1 indicate that B7S1 may be a potential therapeutic target in patients with OvCa who are insensitive to PD-L1/PD-1 inhibition.

The expression level of B7S1 is negatively correlated with the density of TILs (Wang and Wang, 2020). In our study, compared with B7S1^hi^ patients, B7S1^low^ patients displayed decreased frequencies of CD4^+^ T cells and CD4^+^Foxp3^+^ cells, but increased fractions of CD8^+^ TILs and CD8/CD4 ratios. In hepatocellular carcinoma, B7S1 has been found to be upregulated in APCs and to be related to T-cell exhaustion *via* its receptor expressed on early activated CD8^+^ TILs. B7S1 blockade was found to promote CD8^+^ T cell-mediated antitumor immunity in a murine cancer model (Li et al., 2018). Consistent with this, in OvCa, pB7S1R has been detected in CD4^+^ T, CD8^+^ T, NK, and NK T cells. The expression patterns of B7S1 and pB7S1R strongly suggest that B7S1 has an important role in regulating T cells in antitumor immunity in OvCa. Indeed, compared with B7S1^low^ patients, B7S1^hi^ patients displayed more severe immunosuppression in the TME, with a higher percentage of regulatory T cells and a lower fraction of CD8^+^ TILs. Moreover, CD8^+^ TILs isolated from B7S1^hi^ patients displayed characteristic exhausted T-cell phenotypes, including PD-1 expression, decreased CD27 and Ki67 expression, and TNF-α secretion. Thus, through cross-presentation between APCs and CD8^+^ T cells, B7S1 may facilitate the suppression of antitumor immunity in OvCa.

Using publicly available clinical data from TCGA, we focused on the biological functions and underlying mechanisms of *VTCN1* in the TME *via* GO enrichment analysis and GSEA. Several biological processes correlated with immune regulation were identified, including regulation of leukocyte activation, regulation of lymphocyte activation, antigen processing and presentation, regulation of leukocyte cell–cell adhesion, and response to IFN-γ. Importantly, B7S1 can be detected on APCs isolated from OvCa, and high expression of B7S1 is associated with decreased cytotoxic activity of CD8^+^ TILs. Moreover, B7S1 has been reported to inhibit the activation and function of T cells, potently suppressing the proliferation, cytokine production, and cytotoxicity of activated T cells (Sica et al., 2003). When evaluating the underlying mechanisms of *VTCN1*, we found that *VTCN1* was significantly involved in most significant hallmark pathways, including IL-2/STAT5 signaling, p53 pathway, mTORC1 signaling, apoptosis, TNF-α signaling *via* NF-κB, inflammatory response, IFN-γ response, IFN-α response, IL-6/JAK/STAT3 signaling, reactive oxygen species signaling, WNT/β-catenin signaling, and KRAS signaling, in OvCa. These hallmark pathways are related to inflammation, immune regulation, tumor suppressor mutations, reactive oxygen species, cell cycle regulation, and apoptosis. Thus, our findings highlighted the specific associations that may trigger carcinogenesis.

Taken together, our results showed that overexpression of B7S1 in OvCa was positively correlated with antitumor immunosuppression. We also identified a potential immunotherapeutic target for OvCa. Further studies are needed to confirm these results and elucidate the underlying mechanisms.

## Data Availability

The datasets presented in this study can be found in online repositories. The names of the repository/repositories and accession number(s) can be found in the article/Supplementary Material.
